# The diversity of trophoblast cells and niches of placenta accreta spectrum disorders revealed by single-cell RNA sequencing

**DOI:** 10.3389/fcell.2022.1044198

**Published:** 2022-11-07

**Authors:** Jingmei Ma, Yawei Liu, Zhirong Guo, Run Sun, Xinrui Yang, Weiran Zheng, Yongdan Ma, Yin Rong, Hongmei Wang, Huixia Yang, Zhenyu Xiao

**Affiliations:** ^1^ State Key Laboratory of Stem Cell and Reproductive Biology, Institute of Zoology, Chinese Academy of Sciences, Beijing, China; ^2^ Department of Obstetrics and Gynecology, Peking University First Hospital, Beijing, China; ^3^ Beijing Key Laboratory of Maternal Fetal Medicine of Gestational Diabetes Mellitus, Beijing, China; ^4^ Innovation Academy for Stem Cell and Regeneration, Chinese Academy of Sciences, Beijing, China; ^5^ Institute for Stem Cell and Regeneration, Chinese Academy of Sciences, Beijing, China; ^6^ University of Chinese Academy of Sciences, Beijing, China; ^7^ Dushu Lake Hospital Affiliated to Soochow University, Medical Center of Soochow University, Suzhou Dushu Lake Hospital, Suzhou, China; ^8^ School of Life Science, Beijing Institute of Technology, Beijing, China

**Keywords:** placenta, trophoblast, single-cell sequencing, pathogenesis, placenta accreta spectrum

## Abstract

Placenta accreta spectrum disorders (PAS) are severe pregnancy complications that occur when extravillous trophoblast cells (EVTs) invade beyond the uterine inner myometrium and are characterized by hypervascularity on prenatal ultrasound and catastrophic postpartum hemorrhage. The potential mechanisms remain incompletely understood. With single-cell RNA-sequencing analysis on the representative invasive parts and the normal part obtained from the same PAS placenta, we profiled the pathological landscape of invasive PAS placenta and deciphered an intensified differentiation pathway from progenitor cytotrophoblasts (CTBs) to EVTs *via LAMB4* ^*+*^ and *KRT6A* ^*+*^ CTBs. In the absence of the decidua, the invasive trophoblasts of various differentiation states interacted with *ADIRF* ^*+*^ and *DES* ^*+*^ maternal stromal cells. The PAS-associated hypervascularity might be due to the enhanced crosstalk of trophoblasts, stromal cells and vascular endothelial cells. Finally, we presented an immune microenvironmental landscape of invasive PAS. The pathogenesis of PAS could be further explored with current resources for future targeted translational studies.

## Introduction

Placenta accreta spectrum disorders (PAS) represent one of the most severe complications during pregnancy (Silver and Branch, 2018; Matsuzaki et al., 2021). The “spectrum” highlights a range of abnormal placental attachment and invasion to the uterus or other adjacent structures within the same placenta, which can be classified as creta (PC), increta (PI) and percreta (PP) according to the depth of villous tissue invasion into the uterine wall (Silver and Branch, 2018). The consecutive and coexistent pathological features within the same PAS placenta are analogous to a geomorphologic map. Invasive PAS includes PI and PP (Jauniaux et al., 2019; Hecht et al., 2020), which are the major concerns for maternal morbidity and mortality from uterine rupture, catastrophic postpartum hemorrhage, and urinary tract injury (Clark et al., 2008; Creanga et al., 2015; Erfani et al., 2019; Matsuzaki et al., 2021). The effective clinical management for invasive PAS is extremely limited, including elective cesarean hysterectomy or conservation of the uterus by placing an infrarenal aortic balloon to reduce blood loss and then resecting the placenta with the affected portion of the uterine wall. More than 90% of PAS patients have a history of cesarean deliveries (CDs) (Thurn et al., 2016; Jauniaux et al., 2018a). Given the rising rate of CD (Matsuzaki et al., 2021), there has been a 100-fold increase in the incidence of PAS since the 1950s (Wu et al., 2005; Matsuzaki et al., 2021); thus, the clinical significance of this disease has been raised recently.

The pathogenesis of PAS remains poorly characterized. Physical differentiation of extravillous trophoblasts (EVTs) from progenitor cytotrophoblasts (CTBs) is critical for successful pregnancy in humans, as EVTs invade the uterus decidua and remodel spiral vessels into volumetric flow rate-limiting vessels to divert maternal blood into the intervillous space. Coordinately regulated temporal and spatial EVTs were found to be disturbed in PAS, leading to the excessive invasion of EVTs into the myometrium, serosa, and even beyond, as well as dysfunction in the uterine vascular system (Goh and Zalud, 2016; Jauniaux and Burton, 2018), which is reminiscent of cancer-like progression (Goh and Zalud, 2016; Jauniaux et al., 2018b). Despite the progression in the understanding of EVTs invasion in recent decades, the driver of excessive EVTs invasion and hypervascularity remains to be defined.

A history of CD, especially repeat CDs, is associated with the development of large scars, in which the “injured” process of decidualization (Garmi et al., 2011) increases the extent of EVT invasion. Emerging data have revealed that maternal and fetal-derived mesenchymal cells, along with local immune cells, provide a special niche for progenitor CTB maintenance and differentiation. The roles of maternal stromal cells without decidualization in regulating EVT differentiation and in proceeding to vessel remodeling, as well as the immune landscape at the maternal-fetal interface in invasive PAS, have yet to be elucidated.

To understand the complexity of this disease, a comprehensive delineation of the microenvironment involving multiple cell types and their communications in representative invasive PAS placental parts with minimized bias is needed. Single-cell RNA sequencing (scRNA-seq) provides a powerful tool to reveal the cellular and regulatory landscape of both biological processes and disease progression (Puram et al., 2017; van Galen et al., 2019; Welch et al., 2019). Research on the heterogeneity of placental cellular landscapes in invasive PAS and the regulatory cellular and molecular features is thought to hold the key clue to track the potential targets for diagnosis and treatment.

In this study, we employed scRNA-seq and the “within control” comparison on the same invasive PAS placenta, similar to the “cancer and para-cancerous tissue”. With this approach, we focused our analysis on trophoblasts, stromal cells, vessel endothelial cells and immune cells. Among trophoblast cells, we identified two CTB cell types with *LAMB4* ^*+*^ and *KRT6A* ^*+*^ expression, which have not been previously described, revealing the unique cellular heterogeneity in the PAS placenta, which presented intermediate states during the differentiation pathway from primitive CTBs to EVTs. Along with EVTs, the distribution of intermediate CTBs encountered and communicated with maternal stromal cell subtypes, *ADIRF* ^*+*^ and *DES* ^*+*^, surrounding the enhanced vascularity in invasive PAS. The angiogenesis-associated receptor–ligand pairs among intermediate CTBs and EVTs with other maternal stromal cells, as well as the more diversified immune cells, further elaborate the potential mechanism of hypervascularity featured in PAS.

## Materials and methods

### Experimental model and subject details

All placental tissues (Supplementary Table S1) used were collected from patients at Peking University First Hospital with written informed consent. The institutional review boards of Peking University First Hospital approved this study (2019 No. 175). For the case diagnosed as increta (case PAS-1, gravida 4, previous parity 1), the ultrasound profile (Supplementary Figure S1A) met the criteria of International Federation of Gynecology and Obstetrics (FIGO) (Cali et al., 2019) grade 2 (PAS2, at least with two placental lacunae), with the largest lacunae located around the lower part of the front wall (covered 0.87 cm*0.51 cm*0.30 cm), loss of the clear zone, and serosa interruption (approximately 0.42 cm). The scoring system (Zheng et al., 2021) based on risk factors and ultrasonic features of PAS (Supplementary Figure S1A) used to evaluate the severity was more than 10. After antepartum glucocorticoid treatment for fetal lung maturity, selective cesarean section was performed at 34 gestational weeks. According to the FIGO consensus guidelines on PAS disorders involving conservative management (Sentilhes et al., 2018), the PAS placenta was obtained from the removed invaded myometrial tissue, where the invaded myometrial tissue was resected, and myometrial reconstruction was performed. The patient was clinically (Jauniaux et al., 2019) and pathologically (Hecht et al., 2020) diagnosed with invasive PAS (Supplementary Figures S1A-C and Supplementary Table S1).

### Cell isolation from placenta diagnosed with PAS

**FIGURE 1:**
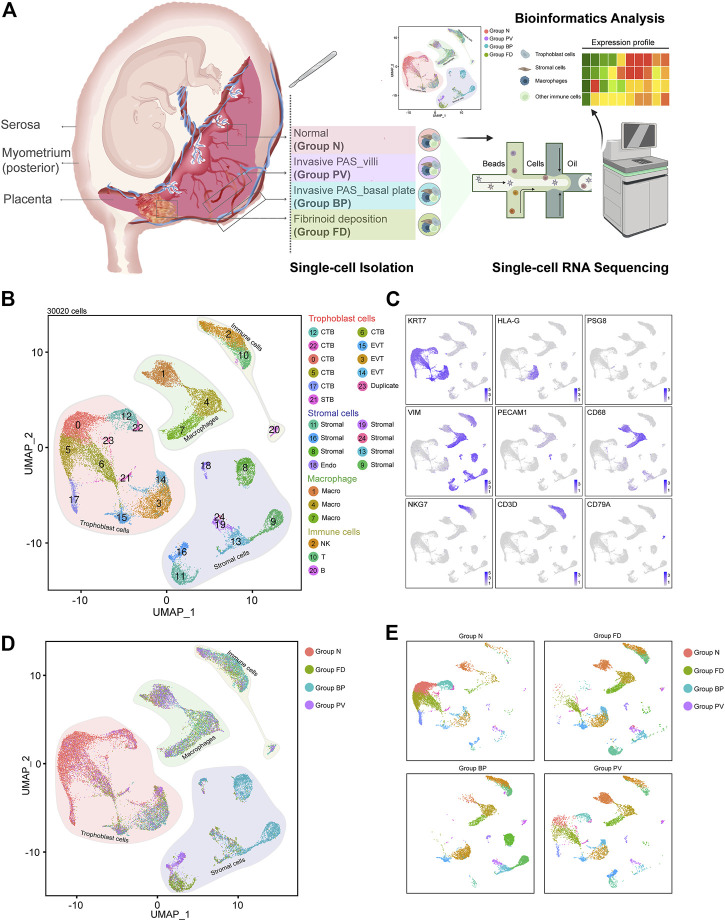
Distinguished cell clusters were classified for the placenta diagnosed as invasive PAS. **(A)** Schematic overview of the workflow of the experimental design in this study, in which four representative parts were obtained and isolated for single-cell RNA sequencing. The four parts were carefully chosen and defined as Group N (N, with villi and basal plate in maternal fetal interface), Group FD (F, adjacent fibrinoid deposition, frequently seen in PAS placenta), Group BP (invasive PAS_basal plate, i.e., the whole uterine wall, where the floating villi were deeply implanted into thin muscles, with inside, outside and sagittal views), and Group PV (dissected from the area of failure detachment of the placenta). Created with BioRender.com. **(B)** UMAP plot showing unbiased clustering of 193,033 cells derived from all four groups. **(C)** UMAP plot showing the expression of the indicated genes. **(D)** UMAP plot of datasets colored, and **(E)** the split UAMP plots by origin of each group from the invasive PAS placenta.

The distribution of lesions in the invasive PAS placenta represented the geomorphologic map concept (Figure 1A and Supplementary Figure S1B). To systematically examine the cellular profiles of the placenta and related decidual tissue in PAS, we performed scRNA-seq analysis on isolated cells from both invasive and normal placental tissues. As the normal group (Group N), the invasive PAS_villi group (Group PV), the invasive PAS_basal plate (Group BP) group and the fibrinoid deposition group (Group FD) coexist in the same PAS placenta, these four parts of the placenta diagnosed with PAS were carefully selected based on macroscopic and microscopic features (Supplementary Figures S1B,C). In Group N, both the villi and basal plate were intact and taken from the normally detached placenta. Group PV included the villi that failed to detach from the uterine myometrium, and Group BP included the whole uterine thin wall, where the villi were deeply implanted. In this way, the combination of Group BP and Group PV served as the invasive PAS-specific lesion. In addition, Group FD was sampled since excessive fibrinoid deposition could frequently be found in the accreta placenta (Jauniaux et al., 2022). These four parts of tissues were enzyme-digested with a protocol from Wang’s laboratory (Liu et al., 2018). The resources could be traced in Table 1.

**TABLE 1:** Resource Identification Initiative.

Reagent or resource	Source	Identifier
Biological samples		
Placenta samples	Peking University First Hospital	N/A
Antibodies		
Anti-human CFD	Abcam	Cat# ab213682
Anti-human CD31	Abcam	Cat# ab28364
Anti-human HLA-G	Proteintech	Cat# 66447-1; RRID:AB_2881816
Anti-COMP	Thermo Fisher Scientific	Cat# PA5-95547; RRID:AB_2807349
Anti-CD36	Cell Sigaling	Cat# 14347; RRID:AB_2798458
Anti-ADIRF	Thermo Fisher Scientific	Cat# PA5-55424, RRID:AB_2638077
Anti-ACTG2	Abcam	Cat#ab231802
Anti-KRT6A	Thermo Fisher Scientific	Cat# PA5-61074, RRID:AB_2640358
Anti-LAMB4	Novusbio	Cat#NBP2-14182
Chemicals, Peptides, and Recombinant Proteins		
10X PBS Buffer	ThermoFisher	Cat# AM9625
Formalin solution	Sigma‒Aldrich	Cat# HT501128
Ethanol	Sinopharm	Cat# 10009218
Xylene	Sinopharm	Cat# 10023418
Paraffin	Leica	Cat# 39601095
DMEM	HyClone	Cat# SH30022.01B
Trypsin	Sigma	Cat# T4799
DNase	Sigma	Cat# 475801
Collagenase, type IV	Gibco	Cat# DN25
Critical Commercial Assays		
Chromium Single Cell 3′ Library & Gel Bead Kit v3.1	10X Genomics	Cat# 1000075
Deposited Data		
10X genomic snRNA-seq datasets	GSA	HRA001965
Software and Algorithms		
Cell Ranger 2.1.1	10× Genomics	https://support.10x genomics.com/singl e-cell-gene- expression/software/downloads/latest
Seurat 4	Hao et al., 2021	R package seurat
Monocle 3	Qiu et al. (2017)	R package monocle

### Library preparation and sequencing

After isolation of cells from the four areas of tissues, a 10 × Genomics Single Cell v2 kit was used for sequencing library preparation per the manufacturer’s protocol. Generally, single cells suspended in PBS were loaded onto a Chromium single-cell controller (10x Genomics) to capture cells, after which captured cells were lysed, and the released RNA was barcoded and then used for sequencing library preparation. Sequencing was performed on an Illumina NovaSeq6000 sequencer performed by CapitalBio, Beijing, China.

### Raw sequencing data processing and quality control

The Cellranger 2.0 pipeline was used to generate gene-cell matrices by mapping to the GRCh38 reference genome. Raw FASTQ files were aligned, filtered and counted with the Cellranger count function. The Cellranger aggr function was used to aggregate and normalize data from different panels of the four groups.

The output of gene-barcode matrix was subjected to further analysis with Seurat packages (version 4.0.1) in R environments. Briefly, the cells detected with less than 200 or more than 7,000 unique genes or with a rate of mitochondria-related genes/all genes over 15% were also discarded to avoid empty droplets, multiplets and dying cells. Data were normalized using the NormalizeData function from Seurat (LogNormalize method using a scale factor of 10,000).

### Sequencing data integration and analysis

After feature selection and scaling of the normalized data, we performed PCA linear dimensional reduction. The first 20 PCs were used to construct the KNN graph with the FindNeighbors function. The Louvain algorithm was performed for clustering the cells with a resolution between 0.2 and 0.8 with the FindClusters functions. After the clusters were identified, differentially expressed gene analysis was performed with the FindAllMarkers function in the Seurat package.

Cell types were annotated based on unsupervised clustering, differentially expressed genes, and well-established marker genes for each cluster. To identify major subtypes (trophoblast, stromal_endo, macro, immune), the expression of *KRT7, HLA-G, PSG5, VIM, PECAM1, CD14, CD68, TRAC, CD3D* and *NCAM1* was evaluated. The subtypes of each major cell type calculated with the FindClusters function were further annotated with the specific marker gene for each subtype calculated by the FindAllMarkers function in Seurat.

Clusters identified in the Seurat package were used for pseudotime analysis with monocle three packages (version 1.0.0) (Trapnell et al., 2014; Qiu et al., 2017). In brief, the expression matrix and metadata derived from Seurat object were first used to build a CellDataSet for the Monocle pipeline with the function new_cell_data_set. Second, the learn_graph function was used to investigate the gene expression changes during the potential trajectory. Finally, we used the function plot_cell to visualize the pseudotime analysis results.

The Velocyto package was used to estimate the RNA velocities of single cells by distinguishing unspliced and spliced mRNAs (La Manno et al., 2018). Briefly, the Python script velocyto. py was used to generate the. loom file and annotate the spliced and unspliced reads for the file derived from the Cellranger output. UMAP coordination of cells derived from Seurat packages was used to project the RNA velocity vectors of the. loom files onto two-dimensional embedding of the UMAP plot.

The cells used for our analysis could be grouped into different categories based on pathological conditions of the placenta. We used the ‘barplot’ function in R to display the cell type dynamics across the different pathological conditions.

We used SCENIC to predict the transcription factor for the cell types identified in our study (Aibar et al., 2017). Briefly, GENIE3 was used to infer the coexpression modules. Then, coexpression modules with false positive predictions were excluded using cis-regulatory motif analyses in RcisTarget. Third, AUCell was used to score all cells for the activity of each regulon. We used the function ‘Heatmap’ in the package complex heatmap to display the top listed regulons for the cell types indicated. The UMAP coordinates of single cells derived from Seurat packages were used to project representative regulons calculated onto two-dimensional embedding of the UMAP plot.

GO analysis for the indicated cell cluster was performed and used to defer the potential functions for the indicated clusters *via* the DAVID website (https://david.ncifcrf.gov/home.jsp) (Huang et al., 2009). Generally, differentially expressed genes between different clusters were calculated with the “FindMarkers” function in Seurat packages, and the list of differentially expressed genes for indicated clusters was further used as the input list to defer their functions. The top 10 most significantly enriched GO terms (Biological Process) or the GO terms (Biological Process) with the most counts are displayed for the indicated cell types.

CellPhoneDB 2.0 was used to calculate the potential ligand–receptor interaction between different cell clusters with parameter thresholds = 0.25 and iterations = 1,000 as previously described (Efremova et al., 2020). The ‘Igraph’ package and other R custom packages were used for visualization.

### Immunohistochemistry staining

For paraffin sectioning, after tissue collection, placental tissues were fixed at 4°C overnight. Tissues were dehydrated with 70%, 85%, 95%, 100% ethanol each for 1 h, and after dehydration, tissues were cleared with xylene. Finally, the tissues were immersed and embedded in paraffin. Paraffin-embedded tissues can be subjected to long-term storage and sectioning.

Paraffin-embedded sections were sliced into 5 μm thick slices and used to bind to clean glass slides. For immunohistochemistry staining, paraffin sections were deparaffinized, hydrated, “epitope-retrieved” and stained with the Wang labs’ protocol as described by Fu et al. (2010). Well-prepared sections were used for imaging with a Leica Aperio VESA8 scanner.

## Results

### The cellular landscape was classified in an invasive PAS placenta

After enzyme digestion, two technical replicates of single-cell suspensions from these four placental parts were subjected to scRNA-seq using the droplet-based 10X Genomic platform. Following rigorous quality control (Supplementary Figure S1D), normalization, and elimination of doublets/multiplets, a total of 30,020 cells were retained for subsequent analysis. We performed unsupervised clustering and projected cells in two dimensions using uniform manifold approximation and projection (UMAP). We identified four major cell types (25 subpopulations) in the merged dataset based on the expression of canonical marker genes, including trophoblasts (*KRT7* ^*+*^ *, PSG8* ^*+*^ *, HLA-G* ^*+*^), stromal cells (*VIM* ^*+*^ *, PECAM1* ^*+*^ *, KRT7* ^*low*^), macrophages (*CD68* ^*+*^), and other immune cells (*CD3D* ^*+*^ *, NKG7* ^*+*^ *, CD79A* ^*+*^) (Figures 1B,C). Different cellular distributions were observed among the four groups; specifically, trophoblasts predominated in Groups N and PV, while Groups FD and BP consisted mainly of stromal cells and immune cells, with distinctive ratios of macrophages to other immune cells (Figures 1D,E and Supplementary Figure S1E). These results unambiguously presented the cellular landscape of the invasive PAS placenta at single-cell resolution and revealed intersample heterogeneity of trophoblast cells, stromal cells, macrophages and other immune cells within the same PAS placenta, indicating various multicellular ecosystems.

### The differentiation pathway from primitive CTBs to EVTs was characteristic of invasive PAS

**FIGURE 2:**
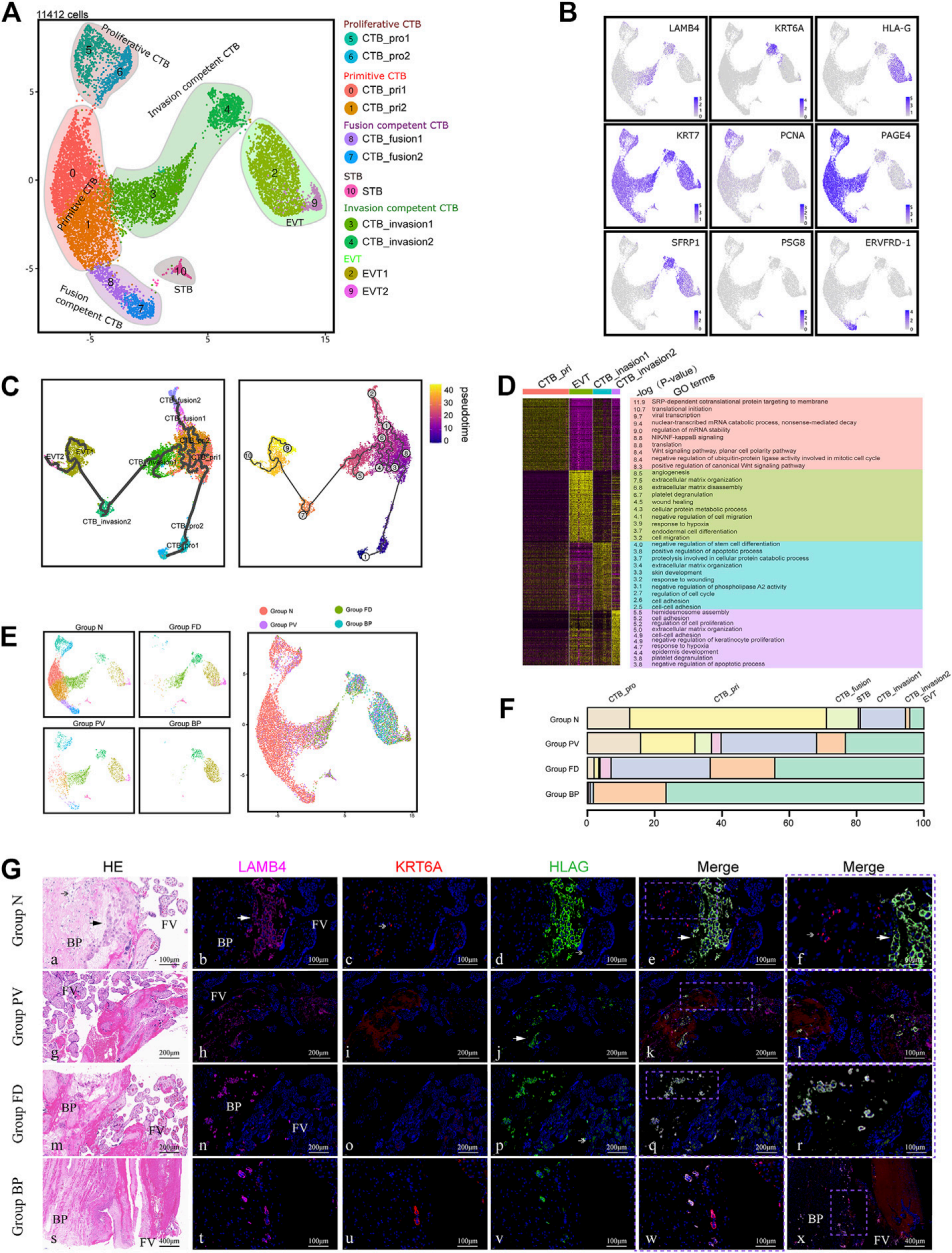
Enhanced differentiation of trophoblasts toward EVT was observed in pathological lesions of the placenta. **(A)** UMAP plot showing the subtypes of trophoblast cells of invasive PAS placenta, with two newly identified EVT competent clusters, CTB_invasion one and 2. **(B)** UMAP feature plots showing the indicated marker genes for the trophoblast clusters, as displayed in Panel 2A. **(C)** UMAP plot displaying the results of pseudotime analysis of all trophoblast cells using Monocle 3. **(D)** GO analysis of four trophoblast cell types along the invasion differentiation pathway, from primitive CTB and CTB_invasion one and two to EVT. **(E)** UMAP plot of trophoblast datasets colored by the origin of each tissue from the PAS placenta. Stacked bar plot showing the unique cellular composition for pathological lesions of PAS placenta. **(F)** Trophoblast ratio comparison of the four groups of PAS placenta. **(G)** The distribution of CTB_invasion 1, 2, and EVT was confirmed with tissues from four groups using markers LAMB4, KRT6A and HLA-G. EVT; CTB_invasion2; BP, basal plate; FV, floating villi.

PAS is associated with extensive EVT invasion. To reveal the potential mechanism, we analyzed trophoblast cells with a focus on differentiation from CTBs to EVTs. We divided 11,412 trophoblasts into three major types. CTBs (*KRT7* ^+^ and *PAGE* ^+^), syncytiotrophoblasts (STBs) (*PSG8* ^+^) and EVTs (*HLA-G* ^+^) were identified based on well-known markers (Figures 2A,B). CTBs could be further divided into eight subtypes: two proliferative CTBs (*PCNA* ^+^), two primitive CTBs (*PAGE* ^+^, *PCNA* ^−^ and *ERVFRD-1* ^-^), two fusion-competent CTBs (*ERVFRD-1* ^+^), and two invasion-competent CTBs (CTB_invasion1, *LAMB4* ^+^ and CTB_invasion 2, *KRT6A* ^*+*^). The latter two clusters were newly identified as potentially specific cell types in PAS placentas.

To explore the differentiation trajectory of the newly annotated trophoblast subclusters, we applied Monocle3 and RNA velocity. Monocle3 demonstrated two pathways extending from proliferative CTBs to EVTs *via* two invasion-competent CTBs or to STB *via* fusion-competent CTBs (Figure 2C). The differentiation trends were further supported by RNA velocity analysis, a useful tool for predicting the future transcriptional state of the cells (Supplementary Figure S2A). Along the differentiation trajectory from CTBs to EVTs, we noticed decreased expression of CTB markers, such as *PAGE4*, and increased expression of EVT markers, such as TAC3. We also found that the expression of *LAMB4*, which is a marker of the intermediate CTB_invasion1, first increased and then decreased during EVT differentiation (Supplementary Figure S2B). Notably, in Group BP, from the fetal to the maternal side, *KRT7* ^+^ trophoblasts sequentially differentiated into CTB_invasion1 (*LAMB4* ^+^), CTB_invasion2 (*KRT6A* ^+^), and EVT (*HLA-G* ^+^) cells, which could be traced in the deep muscular layer near the uterine serosa (Supplementary Figure S2E).

In terms of the functions based on GO analysis, the newly identified intermediate CTB_invasion1 and CTB_invasion2 cells were participated in the regulation of the cell cycle and cell proliferation, indicating their active roles in placental development. Enriched terms such as angiogenesis, extracellular matrix organization, cell migration, as well as negative regulation of cell migration were found in EVT cells (Figure 2D). We also observed that CTB_invasion1 and CTB_invasion2 cells involved in both extracellular matrix organization (ECM), similar to EVTs, while cell-cell adhension was involved by both CTB_invasion1 and 2. Meanwhile, the response to wounding was found in CTB_invasion1, and response to hypoxia was shared by CTB_invasion2 and EVT. This phenomenon raises the possibility that response to wounds originated from previous CS, followed by hypoxia-induced invasion and angiogenesis regulated by CTB_invasion1 and CTB_invasion2 cells may be dominant in PAS placentas.

Scenic analysis was used to further investigate the potential regulatory transcription factors (TFs) that might participate in the differentiation pathway. *FOSL2*, which belongs to the Fos gene family and has been implicated as a regulator of cellular proliferation, differentiation and transformation, was increased in CTB_invasion2 cells. While *ELF3,* as the epithelial cell-specific transcription factor, is a documented tumor suppressor in many epithelial tumors yet displays oncogenic properties in others, was marked in CTB_invasion1 cells. Other distinct important TFs, including *BRCA1, EZH2,* and *STAT4*, were found to be enhanced in proliferative CTBs and CTB_invasion2 cells (Supplementary Figures S2C,D). The functional annotation of these TFs in PAS pathogenesis is worth further exploration.

Different from Group N, the clusters of CTB_invasion1, CTB_invasion2 and EVT were gradually predominant in Groups FD and BP (Figures 2E,F). To validate the differentiation pattern specific to deep invasion of EVTs, we compared the invasive PAS placenta from the second trimester (15 and 23 weeks) with the gestational age-matched placenta, using immunohistochemistry staining with *LAMB4* and *KRT6A*. These controls had previous CS history, with the location of all placentas covered the anterior wall (Supplementary Table S1). The aggregated distribution of CTB_invasion one and two cells along with EVTs in the early onset of PAS was confirmed (Supplementary Figure S2F). In Group normal, the distributions of EVT was patch shaped and confined to the areas surrounded by decidual cells with co-expression of *LAMB4* and *HLA-G*, while CTB_invasion2 was scattered on the way to the deep side (Figure 2G). Intriguing distributing pattern of CTB_invasion2 was found in the floating villi of Group PV, FD and BP, providing candidate targets for future analysis.

With the above findings, the enhanced differentiation pathway from primitive CTBs to EVTs *via LAMB4* ^+^ CTBs and *KRT6A* ^+^ CTBs was identified both in the invasive PAS delivered in the second and third trimesters. We assumed that invasive competent trophoblast cells with an active state of differentiation were closely related to the abnormal deep migration and following vessel remodeling of EVTs in PAS.

### Invasive PAS was associated with distinct stromal cell subtypes

**FIGURE 3:**
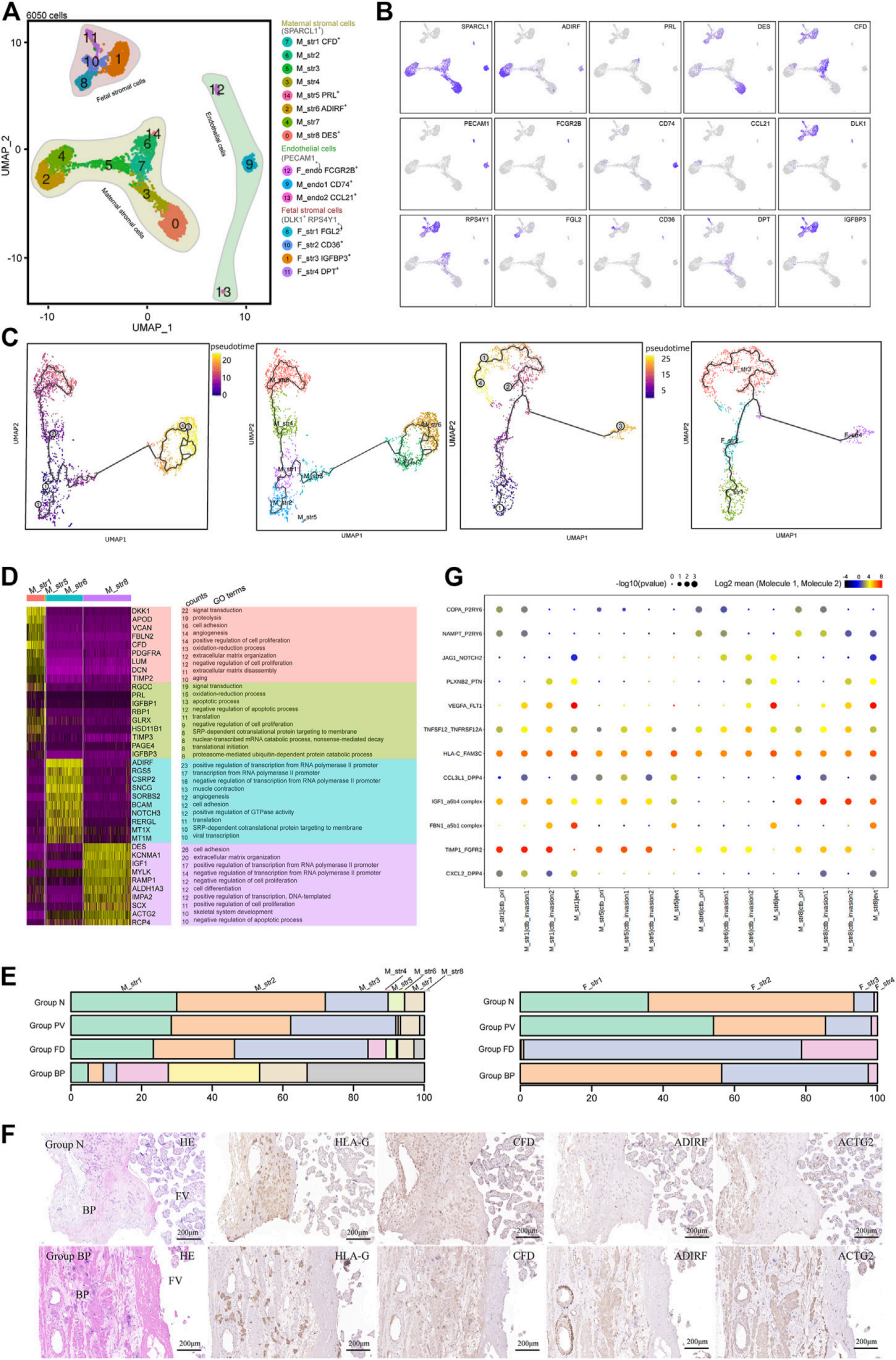
Stromal cells that primarily contribute to the trophoblast microenvironment were analyzed with cellular dynamics. **(A)** UMAP plot showing unbiased subtypes of endothelial cells and maternal and fetal stromal cells for the invasive PAS placenta. **(B)** UMAP feature plots displaying representative marker genes for the stromal cell clusters indicated in Panel 3A. **(C)** UMAP plots showing pseudotime analysis of maternal stromal cells (left panel) and fetal stromal cells (right panel) using Monocle 3. **(D)** GO analysis for the indicated maternal stromal cell clusters. **(E)** Stacked bar plot displaying the dynamics of cell types of maternal (left panel) and fetal (right panel) stromal cell clusters by origin. **(F)** Immunohistochemical staining of tissue from the N and PM groups shows the distribution of maternal stromal cells (*CFD*+, *ADIRF*+ and *DES*+). BP, basal plate; FV, floating villi. **(G)** CellPhoneDB analysis showing the ligand‒receptor pairs among maternal stromal cells and trophoblasts.

PAS progression is mediated by reciprocal interactions among trophoblasts and the surrounding cell types. To analyze the role of stromal cells in PAS pathogenesis, we selected 6,050 stromal cells for subsequent analysis. Three major cell types were annotated based on the expression of canonical marker genes, including maternal stromal cells (*SPARCL1* ^+^), fetal stromal cells (*DLK1* ^+^) and vessel endothelial cells (*PECAM1* ^+^) (Figures 3A,B). Maternal stromal cells were the major types in Group BP, while the other three groups were mainly composed of fetal stromal cells (Supplementary Figures S3A,B).

Maternal stromal cells can be further divided into eight cell clusters in the UMAP (Figure 3A). M_str5 was identified with high expression of *PRL* and *IGFBP1*, representing decidualized stromal cells. M_str6 and eight were two annotated cell clusters with high expression of *ADIRF* and *DES*, respectively. M_str1 is located in the midst of all maternal stromal clusters, with high expression of *CFD*. Other clusters seemed to be the intermediate states of the annotated clusters, displaying the medium level of genes of marker genes (Figure 3B). To investigate the differentiation pathway, Monocle3 (Figure 3C) and RNA velocity assays (Supplementary Figure S3C) were performed, and three differentiation pathways initiated from M_str1 (*CFD* ^+^) to M_str5 (*PRL* ^+^), M_str6 (*ADIRF* ^+^) or M_str8 (*DES* ^+^) were revealed, with decreased CFD levels (Supplementary Figure S3D).

With scenic analysis, distinctive TF expression for each cluster of maternal stromal cells was illustrated (Supplementary Figures S3E,F), as *CREB3L2* and *WT1* were enriched in M_str5 differentiation, while *HOXA13* were involved in M_str8, and *FOXC2* was likely to play a role in the differentiation to M_str6. *HOXA13*, which is a marker of gut primordial posteriorization during development, has been shown to play a crucial role in tumorigenesis of the liver and bladder and in esophageal cancer. *FOXC2* has been reported as the transcription factor that regulate developmental EMT and have a critical role in metastasis, as the highly expressed. *FOXC2*, and the gene products facilitate metastasis in mouse models and human tumors. We performed GO analysis on M_str1, M_str5, M_str6 and M_str8 (Figure 3D). Enriched GO terms in M_str5 were signal transduction, oxidation‒reduction process, and regulation of apoptotic process, while both M_str6 and eight were found to participate in the positive regulation of transcription from the RNA polymerase II promotor. Notably, M_str8 functions to regulate cell adhesion and extracellular matrix organization, and M_str6 promotes angiogenesis. Both clusters might accommodate the invasion of EVTs in the deep uterine wall. Meanwhile, the unique roles of M_str8 in promoting cell differentiation and negatively regulating apoptosis might be important in the pathogenesis of excessive EVT invasion without decidualization.

According to the distribution comparison, the M_str6 and M_str8 subtypes were predominant in Group BP (Figure 3E). The hypothesis was raised that the decline in the decidualization pathway could result in the other two differentiation pathways into M_str6 and M_str eight in invasive PAS. Immunochemical staining with *ADIRF* and *ACTG2* (we used *ACTG2* other than *DES* to respresent M_str8) confirmed the enriched M_str6 and M_str8 subpopulations in Group BP in the absence of M_str5, where deep infiltration of EVTs was found around the vessels, along with *ADIRF* ^+^ stromal cells (Figure 3F). Moreover, as the ligand–receptor pairs revealed by CellPhoneDB analysis, the distinct crosstalk between trophoblasts and maternal stromal cells were angiogenesis-, invasion limited- and differentiation-related, such as *VEGFA-FLT1*, *TIMP1_FGFR2* and *PLXNB2-PTN*, in which M_str5 was superior to M_str6 and M_str8 in invasion limited but not in angiogenesis- and differentiation, indicating the role in regulating trophoblast differentiation and functions in invasive PAS (Figure 3G). In summary, we identified maternal stromal cell subtypes (*ADIRF* ^+^ and *DES* ^*+*^) that formed the frontline to invasive trophoblasts in the absence of the decidua in invasive PAS. Both the analysis of functions and communications were in accordance with their potentials in the niches of invasive EVTs.

Given the important role of floating villi in trophoblast differentiation, we also investigated the distinct profile of fetal stromal cells, which were *DLK1* ^*+*^ and *RPS4Y* ^*+*^, in invasive PAS. The four subtypes were classified as F_str1 (*FGL2* ^+^), F_str2 (*CD36* ^+^), F_str3 (*IGFBP3* ^+^) and F_str4 (*COMP* ^+^) (Figures 3A,B). Pseudotime analysis showed the differentiation trends from F_str1 to F_str3 or F_str4, both *via* F_str2 (Figure 3C and Supplementary Figure S3C). Along the pathway, the expression of *FGL2* was decreased, while the expression of *CD36* was increased from F_str1 to F_str2 and then decreased from F_str2 to F_str3 (Supplementary Figure S3D). TFs that might be involved in regulating differentiation included *TCF21*, *FOX O 3*, *CEBPB,* and *FOSL2* (Supplementary Figures S3E,F).

We further compared the composition of fetal stromal cells. In Group N, F_str2 was dominant, while Group PV consisted of more F_str3 and F_str4. For Group FD, F_str3 and F_str4 were predominant (Figure 3E and Supplementary Figures S3A,B). With GO analysis, the enriched terms in F_str3 included the regulation of cell death, negative regulation of apoptosis, response to mechanical stimulus and hypoxia. The F_str4 subtype was associated with cell adhesion and extracellular matrix organization (Supplementary Figure S3G). The *CD36* ^*+*^ F_str2 and *COMP* ^*+*^ F_str4 subpopulations were identified, as well as an increasing F_str4 in Group PV compared with Group N (Supplementary Figure S3H). Given that turbulent lacunae with high blood flow are typical findings with Doppler imaging, the floating villi in PAS might be influenced by the induced shear stress as a potential mechanical force and then form local fibrinoid deposits, which eventually pomote the differention of CTBs in PAS.

Taken together, both maternal- and fetal-derived stromal cells were found to be unique in PAS lesions, and they formed a microenvironment of stromal cells for both villous and extravillous trophoblasts and might be involved in supporting the unique differentiation of CTBs into invasive EVT cells in invasive PAS.

### Enhanced trophoblast differentiation might be associated with hypervascularity in invasive PAS

**FIGURE 4:**
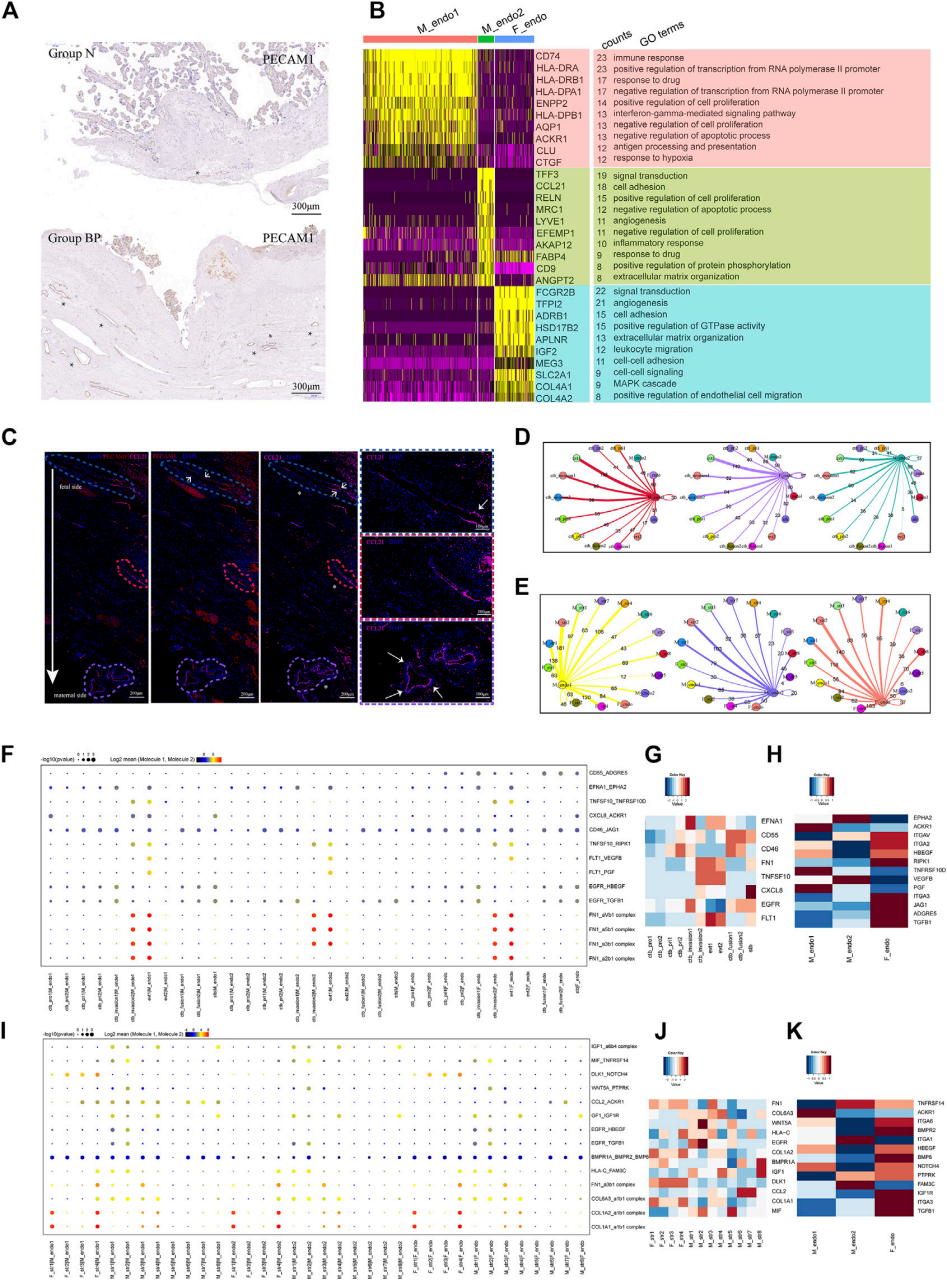
Enhanced differentiation of CTB into EVT might lead to increased angiogenesis in PAS placenta. **(A)** IHC with tissue from groups N and BP showing increased vasculature formation in the BP group. **(B)** GO analysis revealed the difference between the two types of maternal endothelial cells as well as fetal endothelial cells. **(C)** IF with tissue from the BP group showing the distribution of maternal endothelial cells. From the fetal to maternal side, the vessel was gradually dominated by *CCL2*+ endothelial cells. * blood vessels. **(D)** Statistical analysis of the number of ligand‒receptor pairs between endothelial cells and trophoblasts. **(E)** Statistical analysis of the number of ligand‒receptor pairs between the endothelial cells and stromal clusters. **(F)** CellPhoneDB analysis showing the ligand‒receptor pairs among trophoblasts and vessel endothelial cells **(G)**. **(H)** The average expression levels of interacting molecules between trophoblasts and endothelial cells. **(I)** CellPhoneDB analysis showing the ligand‒receptor pairs among stromal cells and endothelial cells **(J)**. **(K)** The average expression levels of interacting molecules between stromal cells and endothelial cells.

The typical feature of invasive PAS is uterovesical hypervascularity, which could induce severe hemorrhage (Supplementary Figure S1A). The hypervascularity in the study was consistent with observations in previous studies. As shown using immunochemical staining with *PECAM1,* the disturbed vascular system in Group BP presented as greater amounts, unevenly distributed along the placenta-muscular junction than those in Group N (Figure 4A). To investigate the underlying regulatory mechanism, maternal vessel endothelial cells, M_endo1 (*CCL21* ^-^ and *PECAM1* ^+^) and M_endo2 (*CCL21* ^+^ and *PECAM1* ^+^), identified from stromal classification (Figures 3A,B), were further analyzed. GO analysis showed that M_endo1 cells were related to the immune response and response to hypoxia, while M_endo2 cells participated in angiogenesis (Figure 4B). The functions of the two clusters were consistent with the distribution, as present in immunofluorescence, in which the M_endo2 cells were increased along the invasion depth deepening from the fetal side to the maternal side, indicating the potential association between EVT invasion and angiogenesis (Figure 4C).

We next performed CellPhoneDB analysis to predict the regulation of vessel endothelial cells. During trophoblast differentiation from primitive CTB to EVT, the numbers of ligand–receptor pairs between the M_endo1 cells and trophoblasts presented an increasing trend, from 60 (CTB_pri1), 95 (CTB_invasion1), and 98 (CTB_invasion2) to 138 (EVT1) pairs (Figure 4D). A similar trend was observed in M_endo2 cells (Figure 4D). Specifically, angiogenesis-related interactions, such as *FLT1-VEGFB* and *FLT1-PGF*, were enriched (Figures 4F–H). In addition, for the interactions between maternal stromal cells and endothelial cells, more ligand–receptor pairs were found in M_str6 and M_str8 cells with endothelial cells than in M_str5 cells (decidual cells) (Figure 4E). The chemotaxis-associated pair *CCL2-ACKR1* was increased between M_str6 and endo1 cells (Figures 4I–K).

Taken together, our results demonstrated the roles of enhanced interactions among location-specific vessel endothelial cells with differentiated trophoblasts and unique maternal stromal subpopulations in promoting angiogenesis in invasive PAS.

### The immune microenvironment at the maternal-fetal interface in invasive PAS is depicted

To characterize the immune landscape in PAS, we analyzed macrophages and other immune cells, NK, T and B cells.

**FIGURE 5:**
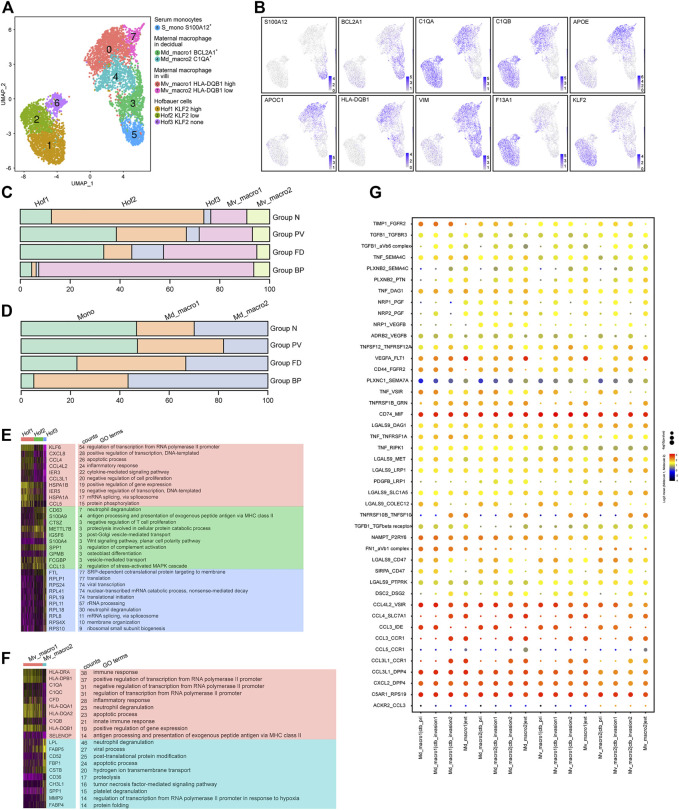
Multiple origins of macrophages at the maternal-fetal interface of the PAS were identified. **(A)** UMAP plot showing reclustering of macrophage cells of human placenta with invasive PAS. **(B)** UMAP feature plots showing representative marker genes for the macrophage clusters shown in Panel 5A, **(C,D)**. Stacked bar plots displaying the dynamics of cell types of floating villi (top panel) and basal plate (bottom panel) macrophage subtypes. **(E)** GO analysis for the indicated Hofbauer cell clusters and maternal macrophages in villi. **(F)** GO analysis for the indicated maternal basal plate macrophage subtypes. **(G)** CellPhoneDB analysis showing the ligand‒receptor pairs among macrophage cells and trophoblasts.

The analysis of 5,505 macrophages identified resulting in eight subtypes (Figures 5A,B), covering two maternal macrophages in villi (Mv_macro1 *HLA-DQB1* ^high^ and Mv_macro2 *HLA-DQB1* ^low^) with high expression of *APOE* and *APOC1*, two maternal macrophages in decidua (Md_macro1 *BCL2A* ^+^ and Md_macro2 *C1QA* ^+^), serum monocytes (S_mono *S100A12* ^+^), and three fetal Hofbauer cells (Hof1 *KLF2* ^high^, Hof2 *KLF2* ^low^ and Hof3 *KLF2* ^none^) with high expression of *F13A1*. Pseudotime analysis (Supplementary Figures S4C-E) showed the potential differentiation pathways from S_mono to Md_macro, and then to Mv_macro, with changing expression of the related markers in each cluster. For Hofbauer cells, RNA velocity analysis showed that Hof1 and Hof3 cells might be derived from Hof2 cells.

For macrophages in villi, Mv_macro1 cells were enriched in Groups FD and PV (Figure 5C), while in the basal plate, Md_macro1 and Md_macro2 cells did not present significant differences among the four groups (Figure 5D). The proportion of maternal serum monocytes was relatively stable in Group N and Group PV (Supplementary Figures S4A,F). Cluster Hof2 represented the major cell type in Group N but was decreased in Group PV (Figure 5C), indicating PAS-induced pathological differentiation among Hofbauer cells. With GO analysis, the shared functions of Hof1 and Mv_macro1 were inflammatory response and antigen processing and presentation (Figures 5E,F). Notably, *MAFB*, a known transcription factor in regulating macrophage differentiation (Basile, 2022), was overexpressed both in Hof2 and Hof1 cells. Distinct TF expression between Hof2 and Hof1 was found, among which *BCLAF1* induces apoptosis when overexpressed (Lee et al., 2012) and might be involved in the differentiation of Hof2 to Hof1 or Hof3 cells (Supplementary Figures S4F,G). We further studied the interactions between invasive trophoblast cells and maternal macrophages by CellPhoneDB, and our results showed significant alterations in the crosstalk between trophoblasts and maternal macrophages (Figure 5G). Specifically, inflammation-related cytokine-receptor interactions involving *CCL3-CCR1*, *CCL3-IDE* and *CCL4L2-VSIR*, along with ligand–receptor pairs contributing to chemotaxis, such as *CXCL2-DPP4* and *CCL3L1-DPP4*, were enriched between maternal macrophages and trophoblasts.

**FIGURE 6:**
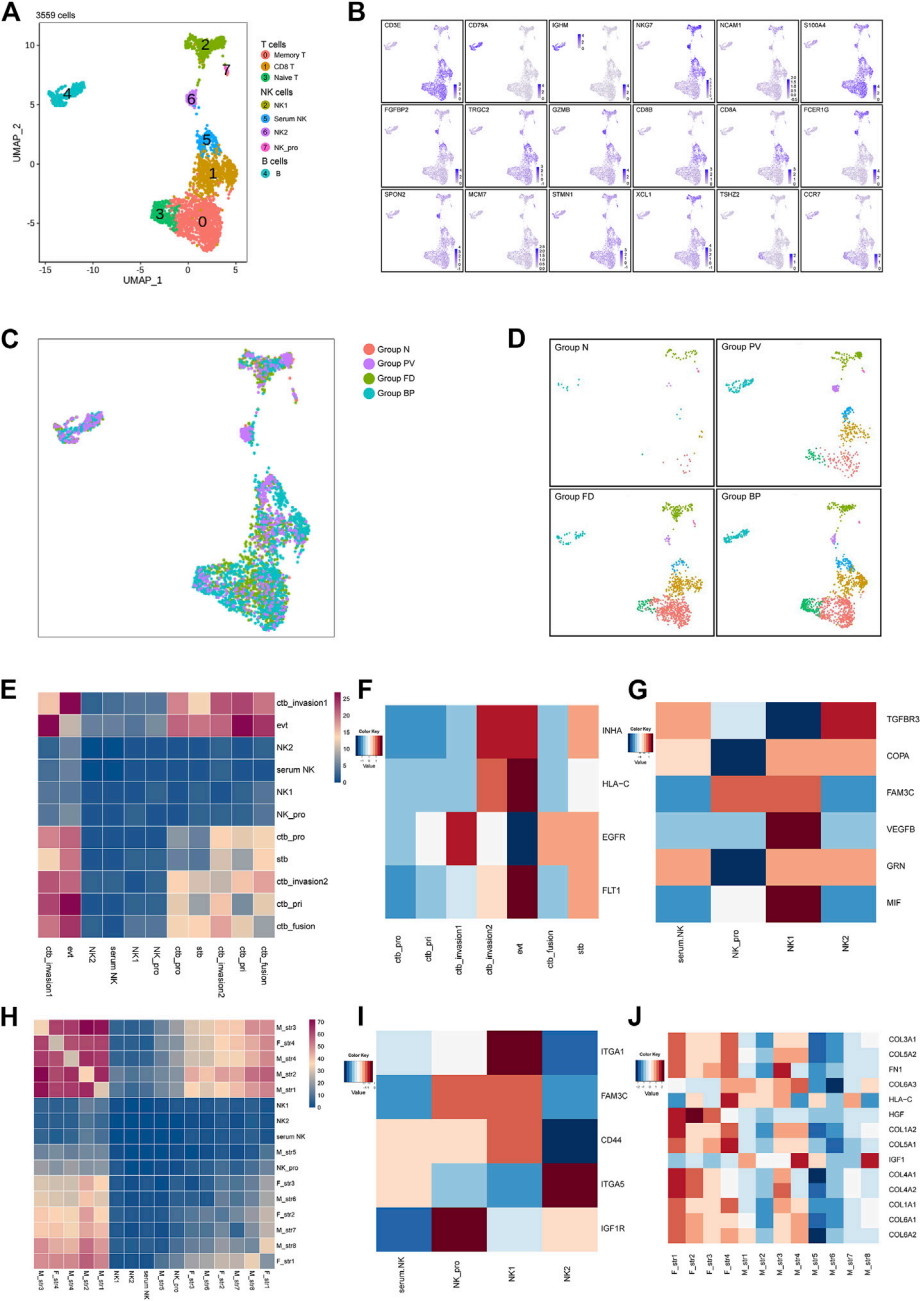
Other immune cells at the maternal-fetal interface for the placenta with PAS. **(A)** UMAP plot showing reclustering of immune cells of PAS placenta. **(B)** UMAP feature plots showing representative marker genes for the cell subtypes shown in Panel 6A. **(C,D)** UMAP plot of other immune cells colored by tissue of origin in the four groups. **(I)**. Split UAMPs displaying other immune subtypes by the origin tissues. **(E,H)** Total number of interactions between immune cells and trophoblasts and stromal subtypes. **(F–J)** Expression levels of ligand‒receptor pairs. The scale is shown beside the plot.

We next examined the 3,559 remaining immune cells, including NK cells, T cells and B cells. Specifically, NK cells (*NKG7* ^high^) were divided into four main subtypes, NK1 (*XCL2* ^*+*^) and NK2 (*FGFBP2* ^*+*^), along with proliferative NK cells and serum NK cells. T cells (*CD3E* ^high^) comprised three subtypes, including CD8 T, naive T (*TSHZ2* ^+^), and memory T (*IL7R* ^+^). Only one B-cell subtype was identified with expression of CD79A (Figures 6A,B). Other immune cells distributed among the four groups are shown in Figures 6C,D, which indicated the enhanced immunological response in pathological groups.

Based on the strength of cellular interactions, the distinctive network at the PAS maternal-fetal interface were characterized. The identified pairs were derived from maternal stromal, trophoblast, and NK clusters interacting with stromal cells or trophoblasts (Figures 6E,H). For trophoblast and immune cell interactions, similar enhanced pairs of FLT1_VEGFB were shared between EVTs and serum NK, NK_pro, NK_1, NK_2, while NK1 showed high expression of *VEGFB*, indicating the regulatory role of NK_1 in angiogenesis by EVTs (Supplementary Figure S5A, Figures 6F,G). There was an increasing trend of active interactions between fetal stromal cells and NK_pro, especially fetal str _1 and fetal str_4, sharing the same ligand_receptor pairs as *COL3A1*_ or *COL1A1*_ expressed by stromal cells and the *a1b1 complex* expressed by immune cells. From the expression level, NK_1 showed *ITGA1*, and NK_2 presented *ITGA5* (Supplementary Figure S5F, Figures 6I,J). The integrin-mediated sigaling pathway was validated by the GO analysis (Supplementary Figure S6), the prominent integrin-mediated signaling pathway presented by NK_2. The active network involving NK cells was consistent with previous findings that angiogenesis during contact interaction of NK cells and endothelial cells in the presence of secretory products of trophoblast cells activated by various cytokines.

Taken together, we clarified distinct immune cells in the invasive PAS-associated groups. We identified eight macrophage subtypes, 4 NK subtypes and 3 T subtypes, which might be involved in PAS disorders. Clarifying the immune landscape and other multicellular interactions in PAS will create new opportunities for the development of targeted therapy in the diagnosis and treatment of PAS. The newly identified differentiation path of Hofbauer cells and the roles of specific TFs in driving distinct immune landscapes in PAS are worth further investigation.

## Discussion

**FIGURE 7:**
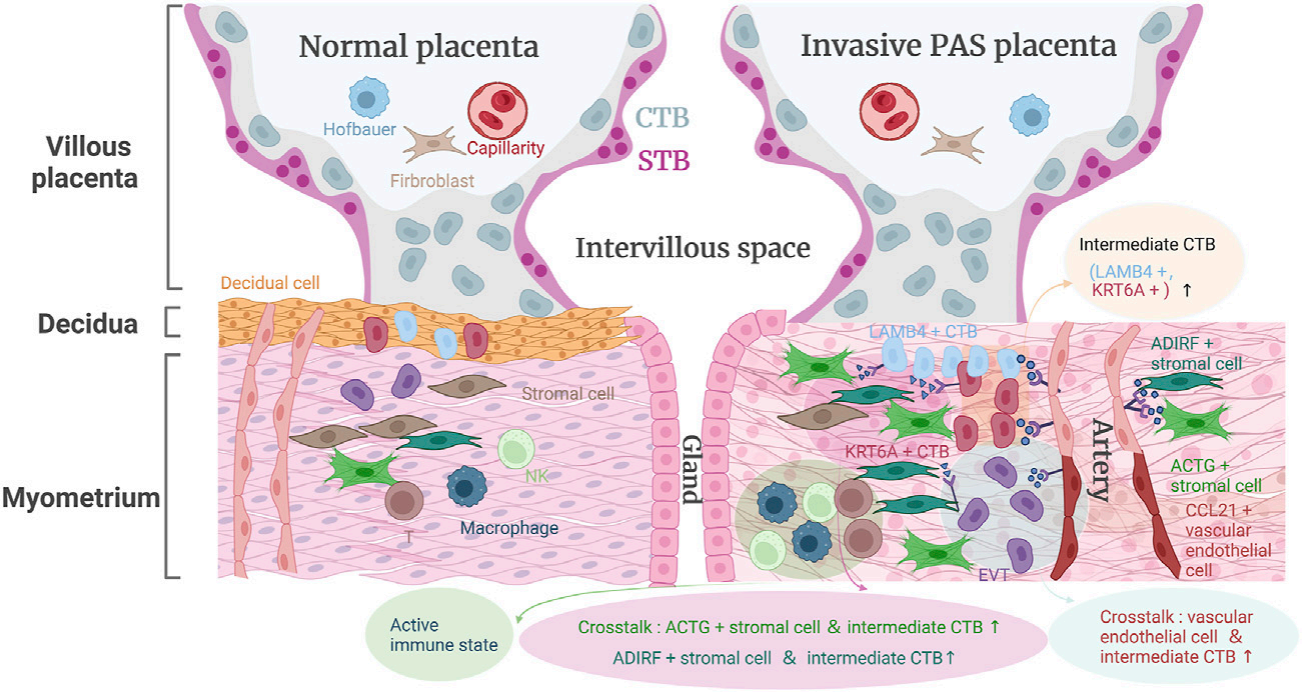
The landscape model of placenta accreta spectrum disorders pathogenesis.

Although collected PAS samples at delivery have enabled accurate correlation of prenatal imaging data with intraoperative features and histopathological findings, the cellular heterogeneity and mechanisms related to PAS remain unknown. Here, we profiled the first single-cell transcriptomic datasets of PAS placenta, which might provide assistance for the early diagnosis and prognosis of PAS (Figure 7).

Our work identified two CTB cell types, namely, *LAMB4* ^+^ and *KRT6A* ^+^ CTBs, with distinct expression profiles that were not described in a previous study, further confirming the presence of these two invasive competent CTB types using histological images in both PAS placenta and stage-matched normal placenta at midgestation, which revealed that the invasive competent CTBs along with EVTs were enriched in the inner myometrium without interposing decidua. In addition, we analyzed noninvasive villi that were taken from the normally detached placenta and provided molecular and cellular evidence for the transition from normal amounts of *LAMB4* ^+^ and *KRT6A* ^+^ CTBs into excess numbers of *LAMB4* ^+^ and *KRT6A* ^+^ CTBs. The scattered invasive competent EVTs together with their close communication with maternal stromal cell subtypes (*ADIRF* ^+^ and *DES* ^+^) and other immune cells (Figure 7) might allow the placental villi to migrate to the serosal surface of the uterus and induce abnormal blood vessels in the uterus through angiogenesis-associated (*FLT1-VEGFA* and *JAG1-NOTCH2*) cell‒cell interactions.

The pivotal regulatory role of maternal decidua in communicating with and regulating placental trophoblast differentiation has been investigated for decades. In the case of PAS, the decidual layer between the villi and the myometrium was completely or partially reduced or defective. Our results indicated that *ADIRF* ^+^ and *DES* ^+^ maternal stromal cells located in deep muscle, in particular, the *DES* ^+^ subpopulation that participated in extracellular matrix organization, cell differentiation, and negative regulation of the apoptotic process, are still capable of controlling invasive trophoblasts in the absence of normal decidua. We further presented an activated immune microenvironment composed of different immune cell subtypes around the PAS placenta in the third trimester. Active NK subtypes were found in PAS pregnancy in the third trimester, recapitulating key features of NK cells in early pregnancy when they comprise the majority of leukocytes (70%) and restricting EVT from excessive invasion.

According to Eric et al. (Jauniaux et al., 2022), when EVTs reaching close proximity to remodel the deep uterine circulation without the normal myometrium structure, the abnormally dilated deep arteries could result high-velocity blood flows entering the intervillous space, the impact of chronic shear stress force then leads to increased fibrin deposition at the uteroplacental interface. Group FD in our study presented the interim EVT differentiation degree. Low oxygen is involved in promoting EVT differentiation during early pregnancy (24), and the thick FD around the aggregated villi might create a hypoxic niche to promote EVT differentiation. Considering our current conception of aggressive EVT invasion in PAS, it will be intriguing to investigate the features of fibrin deposition in PAS in future studies.

In summary, the study profiled a high-resolution single-cell atlas of an invasive PAS placenta for the first time and paved the way for dissecting the molecular and cellular etiologies, with implications in clinical practice.

## Data Availability

The accession number for the sequencing data reported in this paper is GSA: HRA001965. These data have been deposited in the Genome Sequence Archive under project PRJCA008155.
