# Supplementary material for: The diversity of trophoblast cells and niches of placenta accreta spectrum disorders revealed by single-cell RNA sequencing
Source: Front Cell Dev Biol. 2022 Nov 7;10:1044198. doi: 10.3389/fcell.2022.1044198 (PMC9676682; doi:10.3389/fcell.2022.1044198)
Supplement: Supplementary file 1 [file DataSheet1.PDF]

Supplementary Figure 1

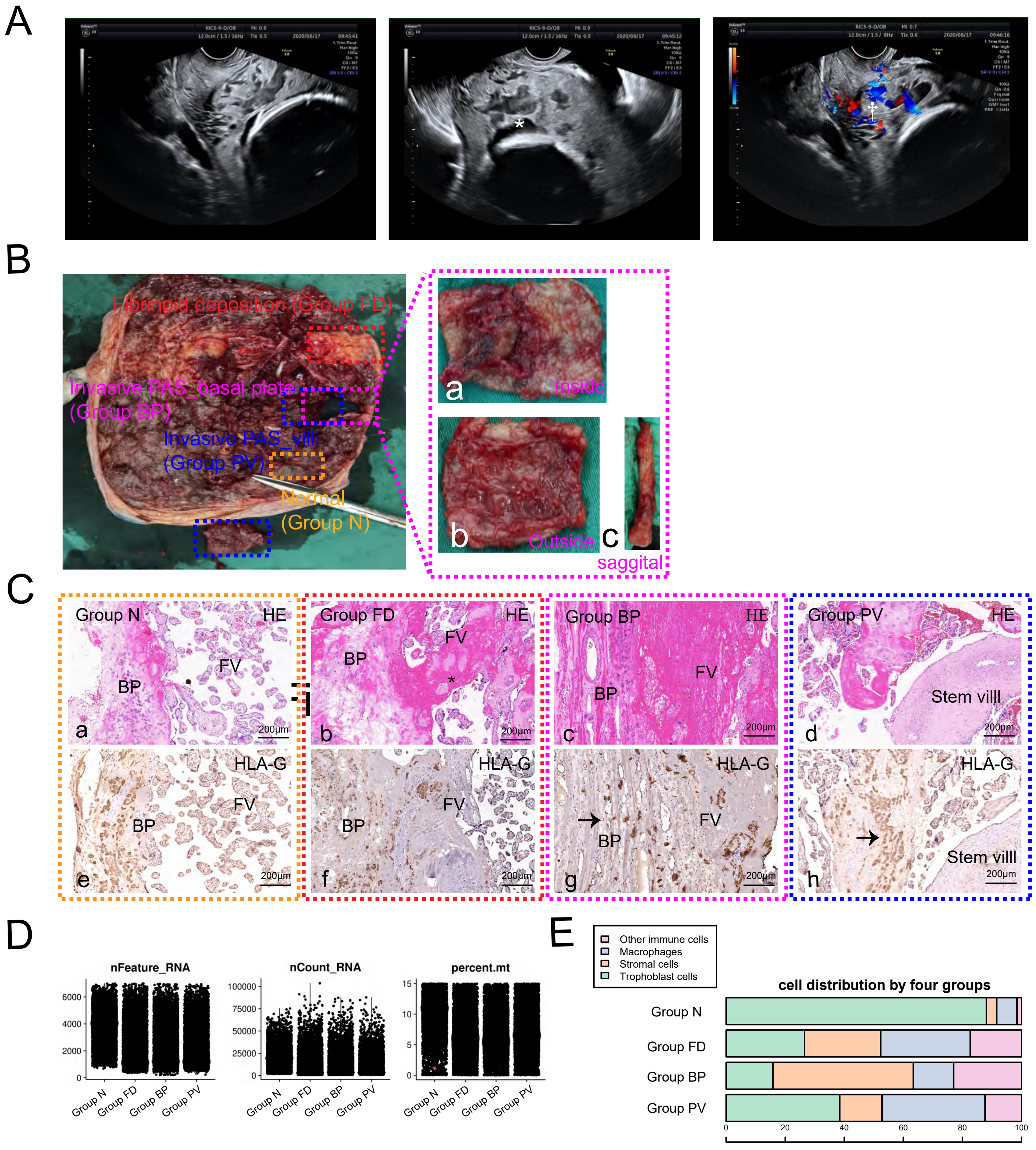

Supplementary Figure 1. Clinical information and quality control of the design. A. Ultrasonic features of invasive PAS patient enrolled in the study. *placental lacunae; † uterovesical hypervascularity. **B.** Sampling and group assignment were based on the macroscopic intraoperative characteristics. **C.** staining of tissue sections of placenta, with the direction from areas of left to right: maternal to fetal side. * showed fibrinoid. Arrow showed EVT with positive HLA-G staining. **D**. Quality of the data based on the origin after quality control. **E.** Barplot displaying cell distribution by four groups used for single-cell RNA-seq analysis.

Supplementary Table1
Demographic information and clinical characteristics of patients enrolled in the study.

Variable	PAS-1	PAS-2	PAS-3	Con-1	Con-2
Maternal age，y	39	42	37	42	35
Gravidity	3	3	2	2	5
Parity	1	1	1	1	1
Gestational age at delivery, wk	34.1	15.0	22.1	14.9	24.1
Previous cesarean deliveries	-	1	1	1	2
In-vitro fertilization	-	-	-	-	-
History of induced abortion	-	Yes	Yes	Yes	Yes
History of other uterine surgery	Yes	-	-	-	-
Previa in current pregnancy	Yes	Yes	-	-	-
Hypothyroidism	-	-	-	-	-
hyperthyroidism	-	-	-	-	-
Diabetes mellitus	Yes	-	-	-	-
Chronic hypertension	-	-	-	-	-
Gestational hypertension or preeclampsia	-	-	-	-	-
Fetal growth restriction	-	-	-	-	Yes
Fetal anomaly	-	-	-	-	-

PAS, placenta accreta spectrum.

Supplementary Figure 2

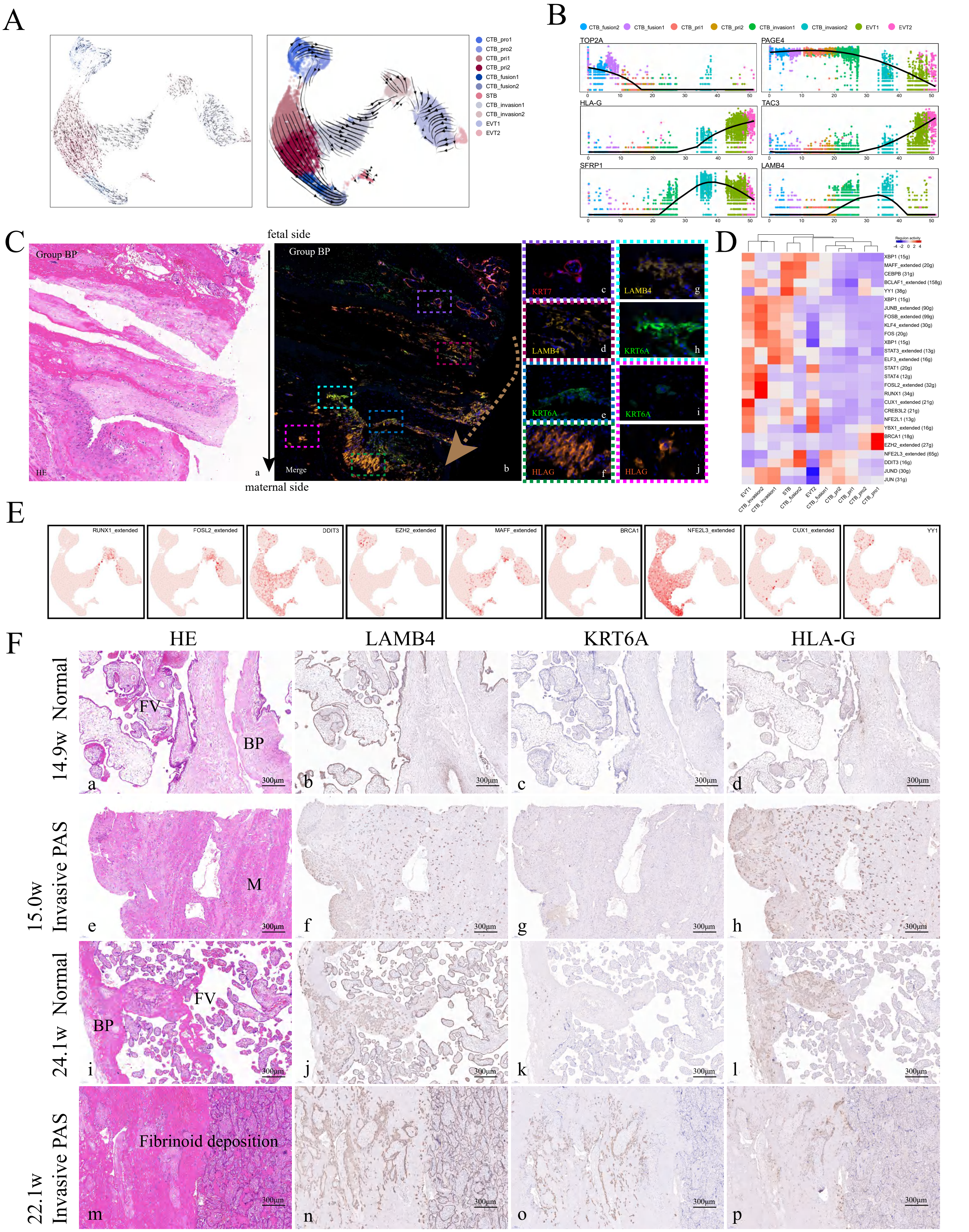

Supplementary Figure 2. Enhanced differentiation of trophoblasts toward EVT was observed in pathological lesions of the placenta. A. The pseudotime analysis of all trophoblast cells was verified by RNA velocity distribution, as UMAP plots showing the RNA velocity for trophoblast cells at the single-cell level (left panel) and as a streamplot (right panel). **B.** The dynamic expression of the indicated genes along the pseudotime of invasion differentiation. **C.** Heatmap displaying the potential master regulators calculated with Scenic for the indicated trophoblast cell types. **D.** UMAP plot showing the activity of the indicated master regulators distributed in the trophoblast cluster. **E.** The distribution of CTB_invasion 1, 2 and EVT was confirmed with tissues from group C, using markers LAMB4, KRT6A and HLA-G. **F.** CTB_invasion 1 and 2 distribution comparison between invasive PAS placentas and normal placentas, using markers LAMB4 and KRT6A with typical PAS placentas delivered at 15.0 and 22.1 gestational weeks. BP, basal plate; FV, floating villi; M, myometrium.

Supplementary Figure 3

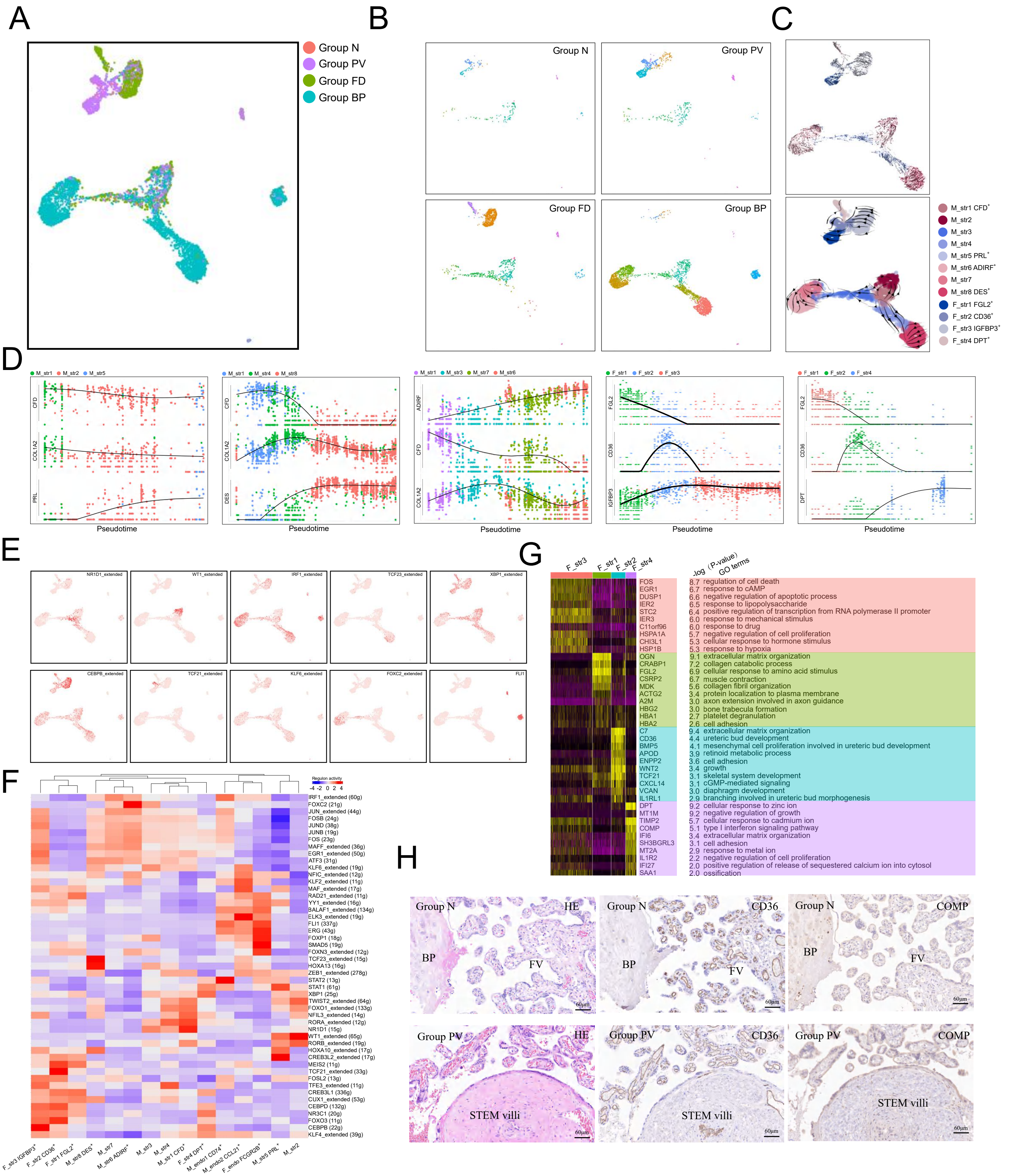

Supplementary Figure 3. Stromal cells that primarily contribute to the trophoblast micro-environment were analyzed with cellular dynamics. **A.** UMAP feature plots displaying representative marker genes for all the stromal cell clusters indicated in Figure 3A. **B.** Stacked bar plots showing the frequencies of the indicated stromal cell types derived from each tissue in PAS placenta. **C.** The pseudotime analysis of all stromal cells was verified by RNA velocity distribution, as UMAP plots showing the RNA velocity at the single-cell level (left panel) and as a stream plot (right panel). **D.** The dynamic expression of the indicated genes on the UMAP plot for pseudotime analysis of stromal cells as shown in Figures 3C and 3D. **E.** UMAP plots showing the activity of the indicated master regulators that might be involved in the functional difference of the indicated stromal cell clusters. **F.** Heatmap displaying the potential master regulators calculated with Scenic for the indicated stromal cell clusters. **G.** GO analysis of the indicated fetal stromal cell clusters. **H.** Immumohistochemical staining with tissue from groups N and PV shows the distribution of fetal stromal cells (CD36+ and COMP+). BP, basal plate; FV, floating villi.

Supplementary Figure 4

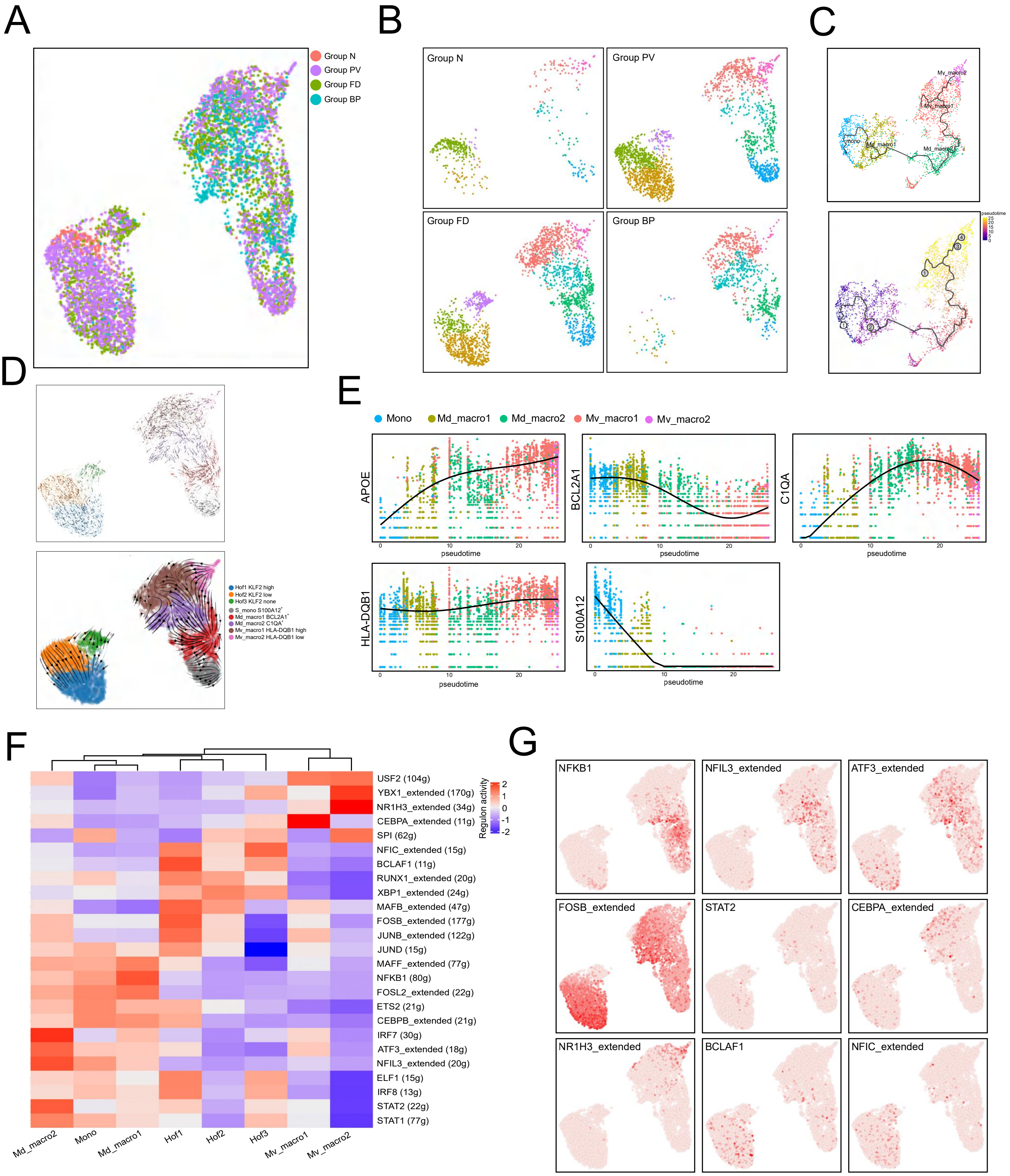

Supplementary Figure 4. Multiple origins of macrophages at the maternal-fetal interface of the PAS were identified.
A. UMAP plot of the datasets of macrophages at the fetal-maternal interface cells colored by the tissue of origin of each group.
B. Split UAMPs displaying macrophage cell clusters by the origin of the group. **C.** UMAP plots showing pseudotime analysis of thematernal and fetal macrophage cells using Monocle 3 (top panel) and annotation of cells along the pseudotime (bottom panel).
D. UMAP plots of RNA velocity for the macrophage cells at the single-cell level (top panel) and as a streamplot (bottom panel).
E. Dynamics of the indicated genes along the pseudotime of the potential differentiation trajectories of macrophage cells.
F. Heatmap displaying the potential master regulators calculated with scenic for the indicated macrophage cell clusters.
G. UMAP plots showing the expression of potential master regulators calculated with scenic for the indicated macrophages clusters.

Supplementary Figure 5

A

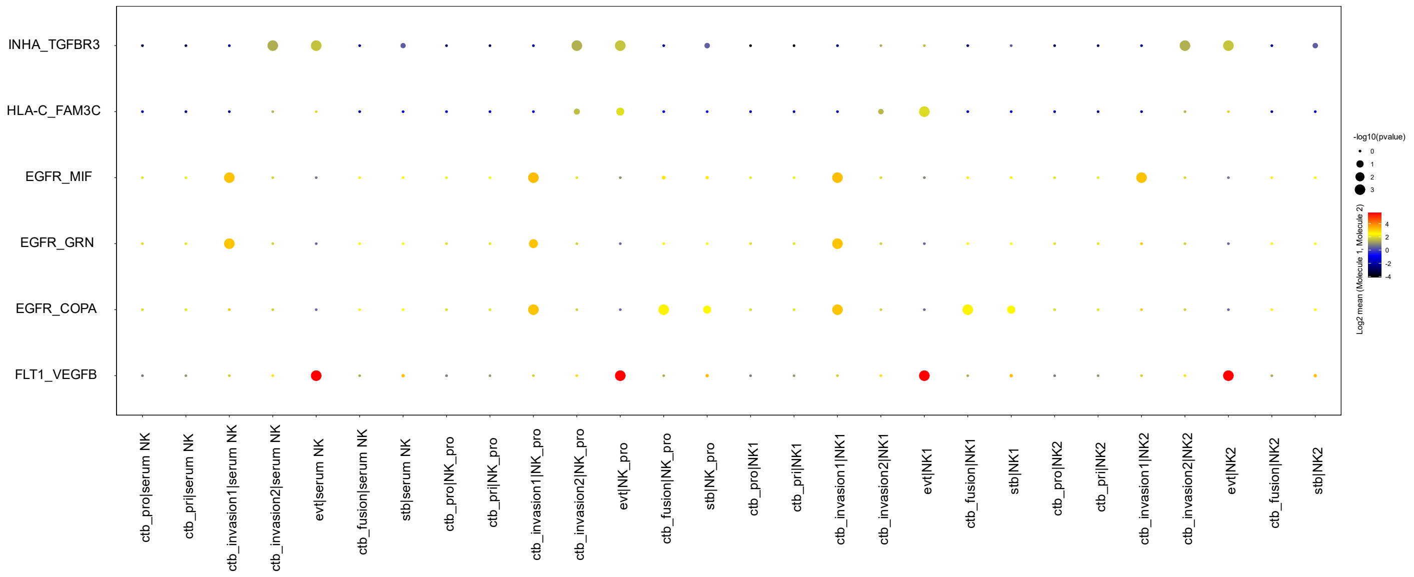

B

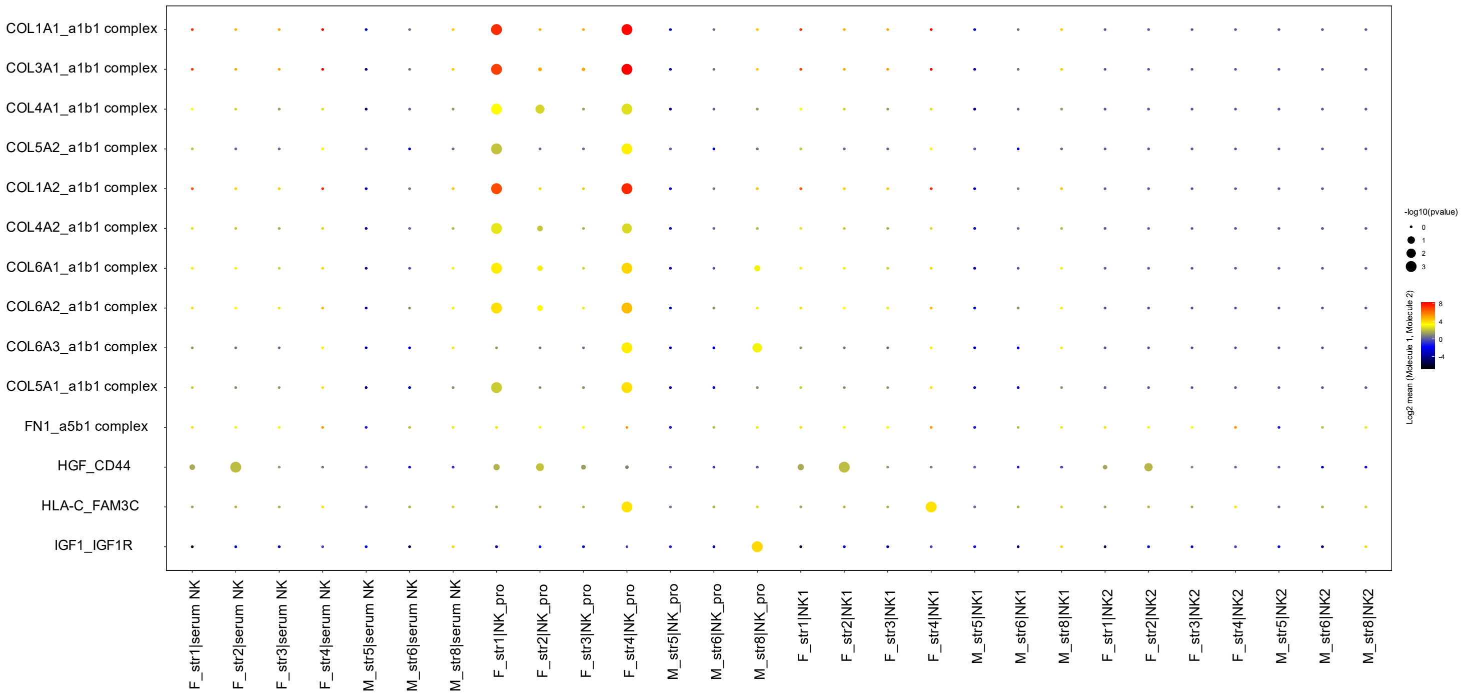

Supplementary Figure 5. Interactions between trophoblasts, stromal cells and other immune cells.
A. Stastistic of number of ligand-receptor pairs between trophoblasts and other immune cells.
B. Stastistic of number of ligand-receptor pairs between stromal cells and other immune cells.

Supplementary Figure 6

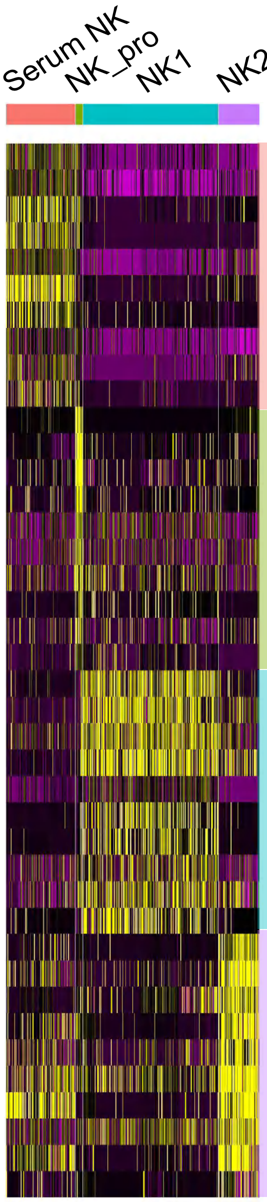

Gene	-log（P-value）	GO terms
TRAC	12.7	adaptive immune response
CD52	12.2	T cell receptor signaling pathway
CD8A	10.1	external side of plasma membrane
CD8B	9.7	alpha-beta T cell activation
CD3D	8.1	T cell activation
CD5	7.3	clathrin-coated endocytic vesicle membrane
TRGC2	7.1	protein binding
ZNF683	6.8	alpha-beta T cell receptor complex
CD6	6.3	cytoplasm
MX2	6.3	extracellular exosome
TCIM	21.6	protein binding
GAS6-AS1	17.2	cytosol
CDT1	14.9	cytoplasm
CDCA7L	14.8	nucleoplasm
GINS2	14.1	nucleus
DTL	10.1	membrane
ENTPD1	6.7	extracellular exosome
CLSPN	6.6	DNA replication
FAM184A	6.4	cell division
MCM7	5.9	double-strand break repair via break-induced replication
WSB2	15.9	protein binding
XCL1	8.0	cytosol
SCX	7.3	apoptotic process
ZNRF1	7.2	nucleus
TYROBP	6.0	negative regulation of cell proliferation
AREG	5.8	response to virus
MAP3K8	5.7	cellular response to tumor necrosis factor
SPRY2	5.3	membrane
XCL2	5.2	negative regulation of apoptotic process
PIK3R1	4.8	positive regulation of phosphoprotein phosphatase activity
FILIP1	6.1	alpha-beta T cell activation
MIR181A2HG	5.5	T cell receptor signaling pathway
CD226	5.4	alpha-beta T cell receptor complex
GPR155	5.2	cytolytic granule
TKTL1	5.1	protein binding
LIM2	4.6	integrin-mediated signaling pathway
PRSS57	4.5	plasma membrane
PXN	4.5	external side of plasma membrane
SIGLEC7	4.2	transmembrane signaling receptor activity
MIR646HG	4.1	immune response

Supplementary Figure 6. GO analysis for the NK cell clusters.
